# No Difference in Behavioral and Self-Reported Outcomes for Simultaneous and Sequential Bilateral Cochlear Implantation: Evidence From a Multicenter Randomized Controlled Trial

**DOI:** 10.3389/fnins.2019.00054

**Published:** 2019-02-20

**Authors:** Véronique J. C. Kraaijenga, Geerte G. J. Ramakers, Yvette E. Smulders, Alice van Zon, Rolien H. Free, Johan H. M. Frijns, Wendy J. Huinck, Robert J. Stokroos, Wilko Grolman

**Affiliations:** ^1^Department of Otorhinolaryngology - Head and Neck Surgery, University Medical Center Utrecht, Utrecht, Netherlands; ^2^Brain Center Rudolf Magnus, University Medical Center Utrecht, Utrecht, Netherlands; ^3^Department of Otorhinolaryngology, Beatrix Hospital, Gorinchem, Netherlands; ^4^Department of Otorhinolaryngology, University Medical Center Groningen, Groningen, Netherlands; ^5^Graduate School of Medical Sciences, Research School of Behavioural and Cognitive Neurosciences, University Medical Center Groningen, Groningen, Netherlands; ^6^Department of Otorhinolaryngology, Head and Neck Surgery, Leiden University Medical Center, Leiden, Netherlands; ^7^Leiden Institute for Brain and Cognition, Leiden University Medical Center, Leiden, Netherlands; ^8^Department of Otorhinolaryngology, Head and Neck Surgery, Radboud University Medical Center, Nijmegen, Netherlands; ^9^Donders Institute for Brain, Cognition and Behaviour, Radboud University Medical Center, Nijmegen, Netherlands; ^10^Causse Ear Clinic, Tertiary Ear Referral Center, Colombiers, France

**Keywords:** bilateral cochlear implantation, sequential, simultaneous, bimodal, QoL, RCT

## Abstract

**Objective:** The primary aim of this study was to longitudinally compare the behavioral and self-reported outcomes of simultaneous bilateral cochlear implantation (simBiCI) and sequential BiCI (seqBiCI) in adults with severe-to-profound postlingual sensorineural hearing loss.

**Design:** This study is a multicenter randomized controlled trial with a 4-year follow-up period after the first moment of implantation. Participants were allocated by randomization to receive bilateral cochlear implants (CIs) either, simultaneously (simBiCI group) or sequentially with an inter-implant interval of 2 years (UCI/seqBiCI group). All sequential patients where encouraged to use their hearing aid on the non-implanted ear over of the first 2 years. Patients were followed-up on an annual basis. The primary outcome was speech perception in noise coming from a source directly in front of the patient. Other behavioral outcome measures were speech intelligibility-in-noise from spatially separated sources, localization and speech perception in quiet. Self-reported outcome measures encompassed questionnaires on quality of life, quality of hearing and tinnitus. All outcome measures were analyzed longitudinally using a linear or logistic regression analysis with an autoregressive residual covariance matrix (generalized estimating equations type).

**Results:** Nineteen participants were randomly allocated to the simBiCI group and 19 participants to the UCI/seqBiCI group. Three participants in the UCI/seqBiCI group did not proceed with their second implantation and were therefore unavailable for follow-up. Both study groups performed equally well on speech perception in noise from a source directly in front of the patient longitudinally. During all 4 years of follow-up the UCI/seqBiCI group performed significantly worse compared to the simBiCI group on spatial speech perception in noise in the best performance situation (8.70 dB [3.96 – 13.44], *p* < 0.001) and localization abilities (largest difference 60 degrees configuration: -44.45% [-52.15 – -36.74], *p* < 0.0001). Furthermore, during all years of follow-up, the UCI/seqBiCI group performed significantly worse on quality of hearing and quality of life questionnaires. The years of unilateral CI use were the reason for the inferior results in the UCI/SeqBiCI group. One year after receiving CI2, the UCI/seqBiCI group performance did not statistically differ from the performance of the simBiCI group on all these outcomes. Furthermore, no longitudinal differences were seen in tinnitus burden prevalence between groups. Finally, the complications that occurred during this trial were infection, dysfunction of CI, facial nerve palsy, tinnitus and vertigo.

**Conclusion:** This randomized controlled trial on bilaterally severely hearing impaired participants found a significantly worse longitudinal performance of UCI/seqBiCI compared to simBiCI on multiple behavioral and self-reported outcomes regarding speech perception in noise and localization abilities. This difference is associated with the inferior performance of the UCI/seqBiCI participants during the years of unilateral CI use. After receiving the second CI however, the performance of the UCI/seqBiCI group did not significantly differ from the simBiCI group.

**Trial Registration:** Dutch Trial Register NTR1722.

## Introduction

Binaural hearing enables a person to differentiate a sound of interest from background noise and localize sounds by using various effects of binaural hearing such as: summation, head shadow, and squelch effect (Dirks, 1969; MacKeith, 1971; Bronkhorst, 1988; Middlebrooks and Green, 1991). There is a wealth of scientific evidence advocating bilateral cochlear implantation (BiCI) over unilateral cochlear implantation (UCI), highlighting that input in both ears instead of one holds evident advantages. In recent years, the difference between BiCI and UCI in adults with severe-to-profound sensorineural hearing loss (SNHL) has been studied thoroughly (Crathorne et al., 2012; Gaylor et al., 2013; van Schoonhoven et al., 2013) evidencing a benefit of BiCI over UCI on speech perception tasks in noise, localization of sounds abilities and quality of hearing (QoH) and quality of life (QoL) improvement. Nonetheless, we believe that a lack of high level concrete evidence such as that derived from a randomized controlled trial (RCT) is much needed to elucidate the BiCI advantage over UCI. Many health care systems such as that in the Netherlands do not reimburse the second cochlear implant in adults due to insufficient proof of societal benefit (cost-utility).

Thus far, there is a lack of overall consensus on whether bilateral cochlear implants should be implanted simultaneously or sequentially. Observational studies have demonstrated advantages of simultaneous BiCI (simBiCI) over UCI as well as sequential BiCI (seqBiCI) over UCI, but no comparative studies exist on the difference between simBiCI versus seqBiCI.

In 2016, our research group published the first results of a RCT comparing outcomes of BiCI to UCI (with or without contralateral hearing aid (HA), e.g., bimodal). It showed conclusive evidence that BiCI patients have superior results over UCI patients on speech perception in noise and localization of sounds using various behavioral and self-reported outcome measures (Smulders et al., 2016; van Zon et al., 2016). UCI patients in that study received a second CI after 2 years of unilateral CI use, enabling researchers to not only investigate the difference between BiCI and UCI (and bimodal), but also evaluate performances between simultaneous BiCI (simBiCI) and sequential BiCI (UCI/BiCI). The results of this cross-sectional comparison demonstrated comparable performances in both groups for almost all outcome measures after 1 year of BiCI experience (Kraaijenga et al., 2017).

It has been reported that short and long-term performance of CI recipients often varies. To date, investigations evaluating long-term outcomes after simBiCI compared with seqBiCI in adult patients are lacking. In the current paper, we present long-term results of 4 years of follow-up using longitudinal analyses that evaluates behavioral outcomes (speech perception and localization), self-reported outcomes (QoL, QoH, and tinnitus outcomes), as well as complications that occurred during the course of this trial.

## Materials and Methods

### Ethical Considerations

This study was approved by the Human Ethics Committees of the Academic Medical Center Amsterdam and consecutively tested for local applicability at all participating centers (University Medical Centers of Utrecht, Maastricht, Nijmegen, Leiden, and Groningen) (NL2466001808), registered in the Dutch Trial Register (NTR1722) and conducted according to the Declaration of Helsinki. Written informed consent was obtained from all participants (Smulders et al., 2016; van Zon et al., 2016; Kraaijenga et al., 2017).

### Study Design and Participants

This RCT compares behavioral and self-reported outcomes of simBiCI to seqBiCI (UCI/seqBiCI group) in adults with severe-to-profound bilateral postlingual SNHL longitudinally during a 4-year follow-up. Data were reported according to the CONSORT statement (Schulz et al., 2010).

Between December 2009 and September 2012, all adults eligible for cochlear implantation by the clinical teams of University Medical Centers Utrecht, Maastricht, Nijmegen, Groningen, and Leiden were assessed for this study’s inclusion and exclusion criteria (Smulders et al., 2016; van Zon et al., 2016; Kraaijenga et al., 2017). The inclusion criteria were: age: 18–70 years; postlingual onset of SNHL; unaided pure-tone average (PTA, mean of 500, 1,000, 2,000 Hertz) ≥70 dB in both ears; duration of severe-to-profound SNHL < 20 years in each ear and a difference in duration of deafness between both ears < 10 years; marginal benefit of HAs, defined as an aided consonant vowel consonant (CVC) phoneme score for both ears of ≤50% at 65 dB sound pressure level (SPL); Dutch as native language; willingness and ability to participate in all scheduled procedures; general health allowing general anesthesia for the duration of potential simBiCI; Dutch health insurance coverage; and agreement to be implanted with Advanced Bionics^®^ implants. The exclusion criteria were: previous CI; abnormal cochlear anatomy; and chronic ear infections; van Zon et al., 2016; Kraaijenga et al., 2017).

### Intervention

**FIGURE 1:**
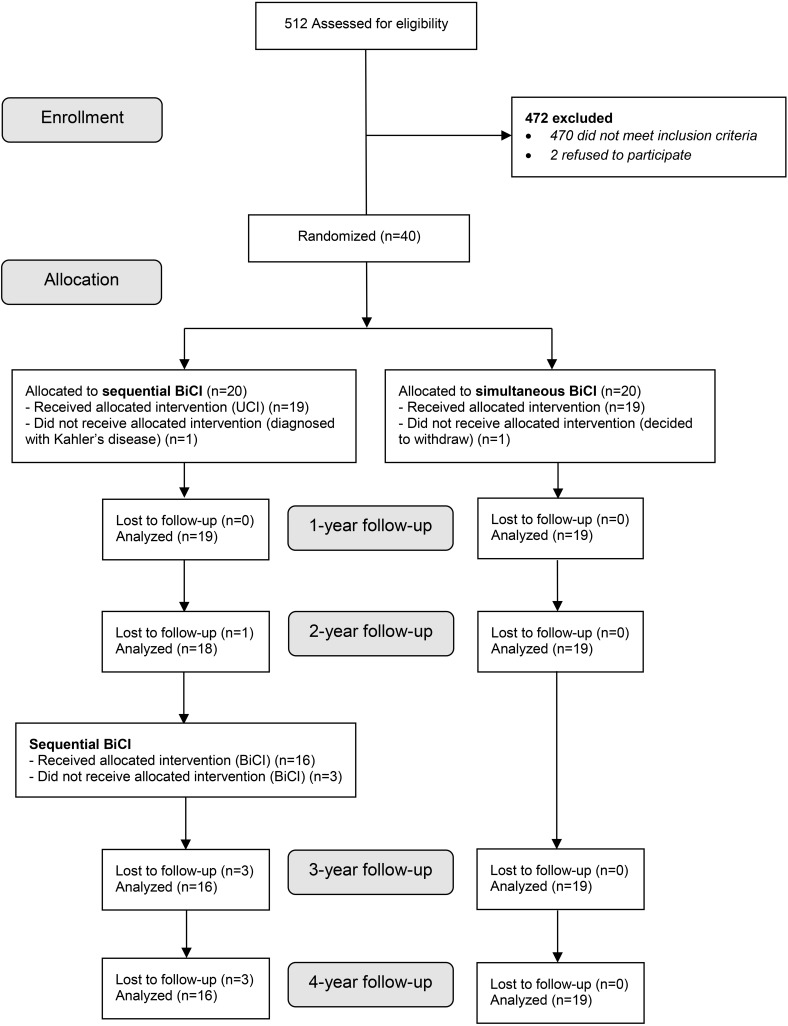
Flowchart of enrollment. simBiCI, simultaneous bilateral cochlear implantation; UCI/seqBiCI, unilateral/sequential bilateral cochlear implantation.

After giving written informed consent and undergoing baseline evaluations, patients were randomly allocated to simBiCI or seqBiCI (UCI/seqBiCI group). It is important to note that in the Netherlands, BiCI is not yet reimbursed in adults. The UCI/seqBiCI group had an inter-implant interval of 2 years (Figure 1). Using a web-based randomization program, a block randomization per center strategy was used to obtain an equal distribution between simBiCI and UCI/seqBiCI groups in all centers (Smulders et al., 2016; van Zon et al., 2016; Kraaijenga et al., 2017). All participants received an Advanced Bionics HiRes90K^®^ implant (Advanced Bionics, Sylmar, CA, United States) coupled with a Harmony processor with HiRes/HiRes120 processing strategies. Participants in the UCI/seqBiCI group were encouraged to keep using a contralateral HA in the first 2 years before sequential implantation.

### Follow-Up

All outcome measures, unless otherwise mentioned below, were evaluated at baseline and after 1, 2, 3, and 4 years of follow-up (Figure 1). The follow up visits lasting approximately 2–2.5 h entailed filling respective questionnaires and behavioral testing. Participants and observers were not blinded for the intervention during evaluations due to the nature of the intervention.

### Behavioral Outcome Measures

Behavioral outcomes included speech perception in noise coming from a source directly in front of the patient (speech and noise from a target located in front of the listener at 0 degrees azimuth), speech intelligibility-in-noise from spatially separated sources (SISSS), localization capabilities and speech perception in quiet. All behavioral outcomes were conducted using the AB-York Crescent of Sound set-up, with horizontally placed loudspeakers in a semicircle around the participant (Smulders et al., 2015). Numbers representing the loudspeaker were shown on monitors below the loudspeakers. In the UCI/seqBiCI group, data were obtained from the ‘participant’s preferred situation’ for the tests conducted before the second implantation. The preferred situation was determined as the situation in which patients performed best, either using the CI (CI1) only or the bimodal condition (CI1 + contralateral HA). In the simBiCI group and for the latter two test moments in the UCI/seqBiCI group, data were gathered from tests performed with both cochlear implants switched on (Smulders et al., 2015; Kraaijenga et al., 2016).

#### Speech Perception in Noise

The primary outcome was speech perception in noise coming from a source directly in front of the patient, measured with the Utrecht-Sentence Test with Adaptive Randomized Roving levels. Dutch VU-98 were presented in noise at 65, 70, or 75 dB SPL (randomly selected). The number of keywords correctly repeated per sentence was scored. Sentences were presented with an initial signal-to-noise ratio (SNR) of +20 dB. If a sentence was scored correct (2 of 3 or 3 of 5 keywords correct), the SNR of the next sentence was decreased by increasing the noise level. Contrarily, if a sentence was scored as incorrect, the SNR was increased. The SNR was altered in steps of 10, 5 and 2.5 dB. The mean SNR of the last 10 sentences was calculated, resulting in a speech reception threshold in noise (SRTn) (Smulders et al., 2015, 2016; Kraaijenga et al., 2016). A lower score reflects better speech perception. An SRTn of 30 dB was considered speech perception in relative silence and was used as a cut-off point for all scores above 30 dB (Kraaijenga et al., 2016).

In the SISSS, also resulting in an SRTn, sentences were presented from 60° azimuth to the left of the patient and noise from 60° azimuth to the right of the patient (S-60 N+60) and vice versa (S+60 N-60) (Kraaijenga et al., 2016). When sounds come from different directions, participants usually have a *best performance situation (BPS)* and a *worst performance situation (WPS)*. A participant’s *BPS* was determined as the situation where speech was presented to the ear with the lowest SNR and noise to the ear with the highest SNR. In a participant’s *WPS*, speech and noise originate from the opposite sides. In the UCI/seqBiCI group before CI2, the *BPS* was defined as the situation in which the target speech was presented to the implanted ear and noise to the non-implanted ear (Kraaijenga et al., 2016, 2017; Smulders et al., 2016; van Zon et al., 2016).

#### Localization Capabilities

For the localization test, participants were instructed to look at the loudspeaker placed directly in front during the entire procedure. A camera was placed in front of the participant and a deviation of the head of the participant was corrected by the observer. Thirty short phrases (“Hello, what’s this?”) were presented randomly at 60, 65, or 70 dB SPL from one of the loudspeakers. The results were percentage of correct responses. The test was performed in three localization conditions: 15° angle azimuth between five loudspeakers, 30° angle azimuth between five loudspeakers, and 60° angle azimuth between three loudspeakers (Smulders et al., 2016; van Zon et al., 2016; Kraaijenga et al., 2017).

#### Speech Perception in Quiet

Speech perception in quiet from a loudspeaker in front of the patient was measured using the standard Dutch CVC test, resulting in a maximum percentage correctly repeated phonemes. This was the only behavioral test which was evaluated at baseline, before randomization.

### Self-Reported Outcome Measures

#### Quality of Life

The QoL questionnaires included the EuroQol five-dimensional questionnaire (EQ-5D), the Health Utilities Index mark 3 (HUI3), a Visual Analog Scale (VAS) on general health, and the Time Trade-off (TTO) (Torrance, 1986; EuroQol Group, 1990; Feeny et al., 2002; Lamers et al., 2005). The EQ-5D contains a thermometer indicating general health state and five dimensions of QoL: mobility, self-care, usual activities, pain/discomfort and anxiety/depression. The result is a single index value for health status: a utility score ranging from -0.33 to 1.00 (EuroQol Group, 1990; Lamers et al., 2005; Ramakers et al., 2016). The HUI3 consists of eight elements of health status. The result is a utility score between -0.36 and 1.00 (Feeny et al., 2002; Ramakers et al., 2016). The VAS on general health contains a thermometer for general QoL, which results in a utility score between 0 and 1 (Ramakers et al., 2016). The TTO is an instrument asking participants whether they are willing to trade expected life years for perfect hearing. The utility is calculated as: utility = (life expectancy – number of years a participant would trade)/life expectancy (Smulders et al., 2016). This question needs good instruction, therefore, it was decided not to let participants answer it independently preoperatively. However, at the 1-,2-,3- and 4-year follow-up moments this information was gathered. For all QoL outcomes, a higher score reflects a better QoL.

#### Quality of Hearing

The QoH questionnaires included the VAS on hearing, the Speech, Spatial and Qualities of Hearing Scale (SSQ) and the Nijmegen Cochlear Implant Questionnaire (NCIQ) (Hinderink et al., 2000; Gatehouse and Noble, 2004). The VAS on hearing contains a thermometer for hearing, which results in a score between 0 and 1 (Ramakers et al., 2016). The SSQ consists of three subdomains. The SSQ1 comprises questions on speech understanding in quiet, in background noise, in reverberant environments and on the telephone. The SSQ2 comprises questions on spatial hearing; identifying directions of sounds and distance approximation, and the SSQ3 comprises questions on the QoH (Gatehouse and Noble, 2004). The results are three subdomain scores ranging from 0 to 10 (Gatehouse and Noble, 2004; Ramakers et al., 2017b). The NCIQ contains six subdomains of hearing: (1) Basic sound perception, (2) Advanced sound perception (in difficult daily listening situations or background noise), (3) Speech production, (4) Self-esteem, (5) Activity limitations, (6) Social interaction (Hinderink et al., 2000). The results are subdomain scores ranging from 0 to 100 (Hinderink et al., 2000; Ramakers et al., 2017b). As this questionnaire is specifically designed for the evaluation after cochlear implantation, this questionnaire was not administered at baseline. For all QoH outcomes, a higher score reflects a greater ability.

#### Tinnitus

The tinnitus questionnaires included the Tinnitus Handicap Inventory (THI) and Tinnitus Questionnaire (TQ) (Newman et al., 1996; McCombe et al., 2001). The THI is a questionnaire regarding tinnitus handicap in daily life. The questionnaire comprises a 12-item functional subscale, an 8-item emotional subscale and a 5-item catastrophic subscale (Newman et al., 1996; McCombe et al., 2001; Ramakers et al., 2017a). The TQ consists of 52 questions on emotional and cognitive distress, intrusiveness, auditory perceptual difficulties, sleep disturbance and somatic complaints (Meeus et al., 2007). Both tinnitus questionnaires were administered to all participants, but could only be completed when a participant experienced tinnitus.

### Sample Size Calculation

Sample size was calculated before the start of the trial using a *T*-test analysis of the primary outcome measure. Fourteen participants in each group were needed to detect a clinically relevant difference of 3 dB in SRTn between groups on the speech perception-in-noise coming from a source in front of the participant test with a standard deviation of 3 dB, an alpha of 0.05 and a power of 80%. Five additional subjects were included per group to compensate for any potential unexpected loss to follow-up (Smulders et al., 2016; van Zon et al., 2016; Kraaijenga et al., 2017).

### Missing Data and Loss to Follow-Up

In case participants were lost to follow-up, analyses were performed with (intention to treat) and without these missing data as a sensitivity analysis.

### Statistical Analysis

Prior to analysis, all data were double-checked by two researchers independently. Patient characteristics were presented as counts, percentages, and medians with interquartile ranges (IQRs).

All outcome measures were analyzed longitudinally (follow-up points 1, 2, 3, and 4 years) via a linear regression analysis with an autoregressive residual covariance matrix (generalized estimating equations type, using a maximum likelihood estimation method). The tinnitus outcomes were analyzed longitudinally via a logistic regression analysis with an autoregressive residual covariance matrix (generalized estimating equations type), as the outcome was dichotomized: the presence of tinnitus burden (yes or no). A participant was considered to experience tinnitus burden when a score higher than 0 was reached on either of the questionnaires.

All models included time (as a categorical variable), group (simBiCI versus UCI/seqBiCI), the interaction between time and group (to study whether the course of scores differed between the study groups) and baseline score of the particular outcome (to adjust for possible baseline differences). Since the TTO and NCIQ were not administered at baseline, the VAS on health and VAS on hearing scores were used as baseline scores. For the speech perception-in-noise and localization tests the CVC phoneme score was used. HA use (yes/no) at baseline (before the study) was the only variable which differed significantly between groups (Smulders et al., 2016; van Zon et al., 2016; Kraaijenga et al., 2017) and for that reason, this variable was added to all models to verify whether it was a possible confounder. Sex and age may have been related to some of the outcomes discussed in this manuscript. If so, sex and age would have also been related to the baseline outcomes. Since we corrected for baseline outcomes, no additional corrections for sex and age were performed. Residuals of the final linear models were checked for normality and showed a normal distribution. To visualize the course of all behavioral and self-reported outcomes for both study groups, all outcome measures were graphed, presenting mean outcome values with standard deviations.

A *p*-value < 0.05 was considered statistically significant. The regression models were generated in SPSS version 22.0 whereas the residue analyses were performed in SAS version 9.4.

## Results

### Participant Characteristics

Between December 2009 and September 2012, 512 patients were assessed for eligibility. Forty participants were randomized and 19 participants were included in each group (Figure 1). Characteristics of participants are described in Table 1 (Smulders et al., 2016; van Zon et al., 2016; Kraaijenga et al., 2017). As previously mentioned, the groups were similar at baseline except for the number of participants using a HA (19 vs. 15).

**Table 1:** Characteristics of participants at time of inclusion in the study.

	UCI/SeqBiCI Median [IQR]	SimBiCI Median [IQR]
Male:Female	11:08	08:11
Age at inclusion (years)	54 [43–64]	52 [36–63]
Duration of severe HL AD (years)	17 [9–33]	16 [11–25]
Duration of severe HL AS (years)	18 [9–35]	16 [11–25]
First CI, right:left	6:13	17:2
PTA AD (dB)	106 [94–111]	106 [89–119]
PTA AS (dB)	108 [93–114]	108 89–120]
CVC phoneme score with hearing aids (%)	44 [29–56]	48 [24–63]
Hearing aid use year 0, yes:no	19:0	15:4
Hearing aid use year 1, yes:no	12:7	Not applicable
Hearing aid use year 2, yes:no	13:5 (1 LTFU)	Not applicable
Treatment hospital		
Utrecht	11	8
Maastricht	4	5
Nijmegen	2	3
Leiden	1	2
Groningen	1	1
Cause of deafness		
Hereditary	7	9
Unknown and progressive	9	6
Sudden deafness	0	2
Head trauma	0	1
Meningitis	2	0
Rhesus antagonism	1	0
Sound exposure	0	1


*BiCI, bilateral cochlear implantation; yrs, years; UCI, unilateral cochlear implantation; Sim, simultaneous; Seq, sequential; AD, auricus dexter; AS, auriculus sinistra; PTA, pure tone average over 1, 2, and 4 kilohertz; CVC, consonant vowel consonant; CI, cochlear implant; LTFU, lost to follow-up; Hz, hertz; IQR, interquartile ranges.*

### Missing Data and Loss to Follow-Up

During the second and third year of follow-up, two participants in the UCI/seqBiCI group withdrew for personal reasons. A third participant was excluded from the UCI/seqBiCI group because of poor performance with the first implant. This participant appeared to have a hearing loss due to rhesus antagonism and was expected not to benefit from a second CI because of this central cause of deafness (Figure 1) (Smulders et al., 2016; van Zon et al., 2016; Kraaijenga et al., 2017).

At year 1, the 15° localization results were missing in one participant in the simBiCI group. A cut-off of 30 dB for speech perception scores was used for one participant in each group. At year 3, the results of the VAS health and hearing were missing in one participant in the simBiCI group and the TTO was missing for another participant in this group. At year 4, the EQ-5D was missing for one participant in the simBiCI group and TTO was missing for another participant in this group.

### Behavioral Outcomes

**FIGURE 2:**
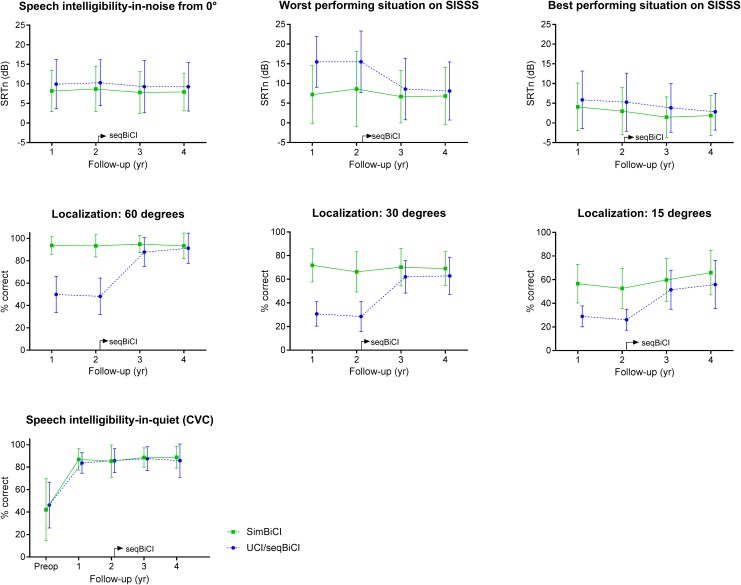
Behavioral outcomes on hearing: a 4-year follow-up. Scores are presented in mean values with an error bar representing the standard deviation. SISSS, speech intelligibility-in-noise from spatially separated sources; WPS, worst performance situation; BPS, best performance situation; CVC, consonant vowel consonant; yr, year; CI, cochlear implant; SimBiCI, simultaneous bilateral cochlear implantation; UCI/seqBiCI, unilateral cochlear implantation/sequential bilateral cochlear implantation group. To improve readability, the results of both groups are presented interleaved, yet follow-up moments were similar in both groups.

Figure 2 shows all behavioral outcomes during the 4 years follow-up for both study groups. Group differences, course per group and difference between follow-up moments per group were analyzed using the previously mentioned linear regression analysis with an autoregressive residual covariance matrix. In the UCI/seqBiCI group, 10 and 11 out of 16 participants used a contralateral HA at year 1 and year 2 respectively.

#### Speech Perception in Noise

Speech perception in noise coming from a source in front of the participant did not differ significantly between UCI/seqBiCI and simBiCI over time (1.99 dB [-1.55 – 5.53], *p* = 0.265) (Table 2). The course of the SRTn did not differ significantly between the two groups and for both groups the SRTns remained stable over time.

**Table 2:** Results from a linear regression analysis with an autoregressive residual covariance matrix (generalized estimating equations type) for all objective outcomes.

	Parameter	Mean	SD	Lower bound 95% CI	Upper bound 95% CI	*P*-value
Speech perception in noise from directly in front (dB)	Treatment	1.99	1.77	-1.55	5.53	0.265
	Year 2	0.49	1.22	-1.91	2.91	0.686
	Year 3	-0.38	1.05	-2.45	1.69	0.718
	Year 4	-0.81	0.80	-2.41	0.78	0.314
	Seq × year 2	-0.18	1.74	-3.63	3.26	0.916
	Seq × year 3	-0.23	1.51	-3.23	2.76	0.877
	Seq × year 4	0.02	1.16	-2.27	2.32	0.986
	CVC baseline	-0.06	0.03	-0.12	0.01	0.076
SISSS: WPS (dB)	Treatment	8.70	2.37	3.96	13.44	**<0.001**
	Year 2	1.51	1.76	-1.97	4.99	0.391
	Year 3	-0.41	1.53	-3.43	2.61	0.787
	Year 4	-0.58	1.19	-2.94	1.77	0.623
	Seq × year 2	-1.50	2.51	-6.47	3.47	0.551
	Seq × year 3	-6.83	2.21	-11.20	-2.45	**0.002**
	Seq × year 4	-7.06	1.71	-10.44	-3.67	**<0.0001**
	CVC baseline	-0.07	0.04	-0.15	0.02	0.113
SISSS: BPS (dB)	Treatment	0.79	1.96	-3.14	4.72	0.688
	Year 2	-1.07	1.40	-3.84	1.70	0.447
	Year 3	-2.60	1.22	-5.01	0.19	**0.035**
	Year 4	-2.68	0.95	-4.56	-0.80	**0.006**
	Seq × year 2	0.34	2.00	-3.62	4.29	0.866
	Seq × year 3	0.48	1.76	-3.01	3.97	0.786
	Seq × year 4	0.60	1.37	-3.31	2.11	0.663
	CVC baseline	-0.012	0.04	-0.20	-0.03	**0.009**
	HA use	7.05	3.42	0.10	14.00	**0.047**
Localization, 15° (% correct)	Treatment	-27.87	5.01	-37.80	-17.94	**<0.0001**
	Year 2	-3.98	4.81	-13.50	5.54	0.410
	Year 3	3.21	4.54	-5.77	12.19	0.481
	Year 4	9.09	3.96	1.23	16.94	**0.024**
	Seq × year 2	1.12	6.82	-12.39	14.60	0.870
	Seq × year 3	19.64	6.53	6.72	32.56	**0.003**
	Seq × year 4	17.85	5.63	6.69	29.01	**0.002**
	CVC baseline	0.05	0.07	-0.09	0.19	0.493
Localization, 30° (% correct)	Treatment	-41.09	4.56	-50.12	-32.06	**<0.0001**
	Year 2	-5.45	4.36	-14.06	3.16	0.213
	Year 3	-1.53	4.09	-9.63	6.56	0.709
	Year 4	-2.25	3.53	-9.27	4.77	0.526
	Seq × year 2	3.24	6.21	-9.02	15.51	0.602
	Seq × year 3	34.20	5.93	22.48	45.92	**<0.0001**
	Seq × year 4	35.73	5.07	25.67	45.79	**<0.0001**
	CVC baseline	0.01	0.07	-0.12	0.14	0.905
Localization, 60° (% correct)	Treatment	-44.45	3.88	-52.15	-36.74	**<0.0001**
	Year 2	-0.35	3.66	-7.58	6.87	0.923
	Year 3	1.23	3.40	-5.50	7.96	0.719
	Year 4	-0.13	2.89	-5.89	5.62	0.963
	Seq × year 2	-1.37	5.21	-11.67	8.93	0.793
	Seq × year 3	38.26	4.93	28.52 –	48.01	**<0.0001**
	Seq × year 4	43.04	4.15	34.78 –	51.29	**<0.0001**
	CVC baseline	0.14	0.06	0.02 –	0.26	**0.020**
CVC score (%)	Treatment	-3.72	3.34	-10.33 –	2.88	0.267
	Year 2	-1.47	3.34	-8.07 –	5.12	0.659
	Year 3	1.79	3.34	-4.81 –	8.39	0.536
	Year 4	2.24	3.44	-4.54 –	9.04	0.514
	Seq × year 2	3.56	4.75	-5.84 –	12.95	0.455
	Seq × year 3	2.14	4.83	-7.40 –	11.69	0.658
	Seq × year 4	-0.13	4.90	-9.81 –	9.55	0.979
	CVC baseline	0.14	0.04	0.06 –	0.21	**<0.001**


*Reference treatment group = simBiCI; reference year = year 1. The final model contained the following variables: time + treatment + time × treatment + baseline CVC phoneme score. In case there was a confounding role for hearing aid use at baseline, this variable was included in the final model as well. SISSS, speech intelligibility-in-noise from spatially separated sources; CVC, consonant vowel consonant; HA, hearing aid; SimBiCI, simultaneous bilateral cochlear implantation; seq, unilateral cochlear implantation/sequential bilateral cochlear implantation group. Bold value means statistical significance.*

In the *WPS* of the SISSS test, the UCI/seqBiCI group performed significantly worse over time: 8.70 dB [3.96 – 13.44], *p* < 0.001. A significant improvement was seen in the UCI/seqBiCI group after receiving CI2 (year 3 (seqBiCI) vs. year 1 (UCI); -6.83 dB [-11.20 – -2.45], *p* = 0.002). In the *BPS* of the SISSS test however, no difference between groups over time was found, yet a significant improvement was seen in the simBiCI group after years 3 and 4 compared to year 1 (year 4 vs. year 1: -2.68 dB [-4.56 – -0.80], *p* = 0.006). HA use at baseline was a significant confounder for the SISSS *BPS*, and therefore the final model was corrected for HA use.

#### Localization

The largest differences between groups were seen on the localization tests over time, for example in the 60 degrees configuration: the scores of UCI/seqBiCI were significantly lower (-44.45% [-52.15 – -36.74], *p* < 0.0001) than the scores of the simBiCI group over time. The UCI/seqBiCI group showed a significant improvement after receiving CI2, which is most evident between year 4 and year 1: 43.04% [34.78 – 51.29], *p* < 0.0001. The direction and significance of the results of the localization tests in 15 and 30 degrees configurations did not differ from the 60 degrees results.

#### Speech Perception in Quiet

The CVC phoneme scores did not differ significantly between groups over time. Also, the course of these scores did not differ significantly between groups and for both groups the scores were stable over time.

### Self-Reported Outcomes

#### Quality of Life Outcomes

**FIGURE 3:**
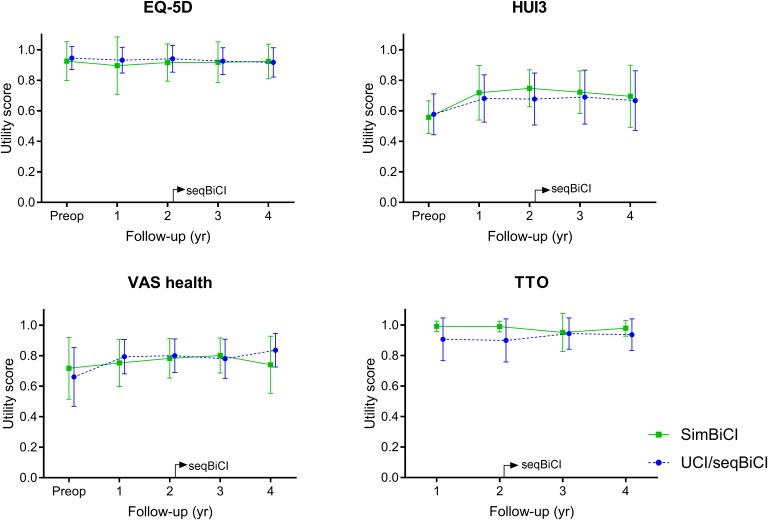
Self-reported outcomes on quality of life: a 4-year follow-up. Scores are presented in mean values with an error bar representing the standard deviation. EQ5D, Dutch EuroQol-5D; HUI3, Health Utilities Index 3; VAS, Visual Analog Scale; TTO, Time Trade-off; yr, year; CI, cochlear implant; SimBiCI, simultaneous bilateral cochlear implantation; UCI/seqBiCI, unilateral cochlear implantation/sequential bilateral cochlear implantation group. To improve readability, the results of both groups are presented interleaved, yet follow-up moments were similar in both groups.

Figure 3 shows the QoL outcomes preoperatively and during the 4 years of follow-up for both study groups. The EQ-5D, HUI3 and VAS general health scores did not differ significantly between the UCI/seqBiCI and simBiCI group over time (Table 3). Also the course of these scores did not differ between groups and for both groups the EQ-5D, HUI3 and VAS general scores remained stable over time.

**Table 3:** Results from a linear regression analysis with an autoregressive residual covariance matrix (generalized estimating equations type) for quality of life outcomes.

	Parameter	Mean	Standard deviation	Lower bound 95% CI	Upper bound 95% CI	*P*-value
EQ-5D	Treatment	0.023	0.031	-0.038	0.083	0.459
	Year 2	0.020	0.030	-0.038	0.079	0.495
	Year 3	0.023	0.028	-0.033	0.079	0.425
	Year 4	0.034	0.025	-0.016	0.083	0.178
	Seq × year 2	-0.009	0.042	-0.093	0.074	0.825
	Seq × year 3	-0.021	0.041	-0.102	0.061	0.618
	Seq × year 4	-0.038	0.036	-0.108	0.033	0.296
	EQ-5D baseline	0.688	0.097	0.493	0.883	**<0.0001**
HUI3	Treatment	-0.011	0.050	-0.112	0.089	0.821
	Year 2	0.030	0.046	-0.060	0.120	0.510
	Year 3	0.005	0.042	-0.079	0.088	0.911
	Year 4	-0.022	0.035	-0.090	0.046	0.525
	Seq × year 2	-0.034	0.065	-0.162	0.095	0.603
	Seq × year 3	0.012	0.061	-0.109	0.133	0.848
	Seq × year 4	0.019	0.050	-0.081	0.119	0.711
	HUI3 baseline	0.520	0.164	0.189	0.850	**0.003**
	HA use	-0.172	0.068	-0.308	-0.036	**0.014**
VAS health	Treatment	0.053	0.040	-0.026	0.132	0.186
	Year 2	0.030	0.035	-0.039	0.099	0.394
	Year 3	0.048	0.032	-0.015	0.112	0.135
	Year 4	-0.012	0.025	-0.062	0.038	0.647
	Seq × year 2	-0.021	0.050	-0.120	0.078	0.672
	Seq × year 3	-0.062	0.046	-0.154	0.029	0.179
	Seq × year 4	0.053	0.037	-0.020	0.126	0.155
	VAS health baseline	0.210	0.077	0.054	0.365	**0.009**
TTO	Treatment	-0.078	0.031	-0.140	-0.017	**0.013**
	Year 2	-0.002	0.030	-0.061	0.057	0.958
	Year 3	-0.040	0.029	-0.096	0.017	0.169
	Year 4	-0.012	0.024	-0.060	0.036	0.627
	Seq × year 2	-0.005	0.043	-0.089	0.079	0.913
	Seq × year 3	0.084	0.041	0.003	0.165	**0.043**
	Seq × year 4	0.047	0.035	-0.023	0.117	0.183
	VAS health baseline	0.124	0.053	0.017	0.231	**0.025**


*Reference treatment group = simBiCI. Reference year = year 1. The final model contained the following variables: time + treatment + time × treatment + baseline score. In case there was a confounding role for hearing aid use at baseline, this variable was included in the final model as well. EQ-5D, EuroQol five-dimensional questionnaire; HUI3, the Health Utilities Index mark 3; HA, hearing aid; VAS, Visual Analog Scale on general health; TTO, Time Trade-off; SimBiCI, simultaneous bilateral cochlear implantation; seq, unilateral cochlear implantation/sequential bilateral cochlear implantation group. Bold value means statistical significance.*

The TTO score was significantly lower in the UCI/seqBiCI group compared with the simBiCI group over time (-0.078 [-0.140 – -0.017], *p* = 0.017). A significant improvement was seen in the UCI/seqBiCI group after receiving CI2 (year 3 vs. year 1: 0.084 [0.003- 0.165], *p* = 0.017). HA use was a significant confounder for the HUI3, and therefore the final model was corrected for HA use.

#### Quality of Hearing Outcomes

**FIGURE 4:**
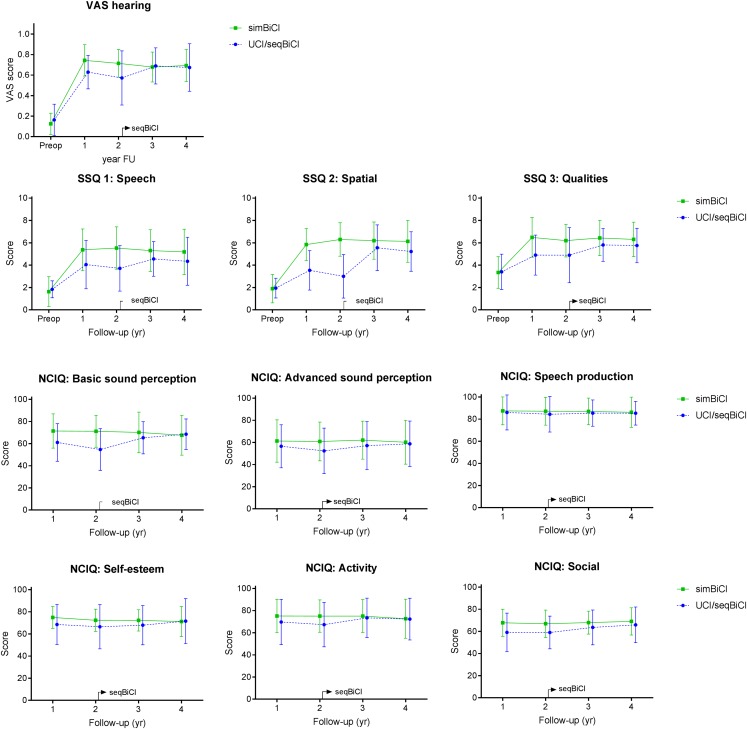
Self-reported outcomes on quality of hearing: a 4-year follow-up. Scores are presented in mean values with an error bar representing the standard deviation. VAS, Visual Analog Scale; SSQ, Speech, Spatial and Qualities Hearing Scale; NCIQ, Nijmegen Cochlear Implant Questionnaire; yr, year; CI, cochlear implant; SimBiCI, simultaneous bilateral cochlear implantation; UCI/seqBiCI, unilateral cochlear implantation/sequential bilateral cochlear implantation group. To improve readability, the results of both groups are presented interleaved, yet follow-up moments were similar in both groups.

Figure 4 shows the QoH outcomes preoperatively and during the 4 years of follow-up for both study groups. The VAS hearing scores differed significantly between the UCI/seqBiCI and simBiCI group over time (-0.12 [-0.24 – -0.01], *p* = 0.036) (Table 4). The course of these scores did not differ between groups. The scores in the UCI/seqBiCI group did not improve significantly after receiving CI2.

**Table 4:** Results from a linear regression analysis with an autoregressive residual covariance matrix (generalized estimating equations type) for quality of hearing outcomes.

	Parameter	Mean	Standard deviation	Lower bound 95% CI	Upper bound 95% CI	*P*-value
VAS hearing	Treatment	-0.122	0.057	-0.236	-0.008	**0.036**
	Year 2	-0.029	0.052	-0.132	0.074	0.581
	Year 3	-0.056	0.048	-0.152	0.040	0.249
	Year 4	-0.050	0.039	-0.127	0.027	0.198
	Seq × year 2	-0.028	0.075	-0.175	0.120	0.710
	Seq × year 3	0.128	0.070	-0.010	0.266	0.069
	Seq × year 4	0.107	0.056	-0.005	0.220	0.060
	VAS hearing baseline	0.201	0.166	-0.132	0.534	0.232
SSQ1	Treatment	-1.524	0.533	-2.591	-0.458	**0.006**
	Year 2	0.161	0.356	-0.542	0.865	0.651
	Year 3	-0.063	0.304	-0.665	0.538	0.835
	Year 4	-0.183	0.225	-0.630	0.263	0.418
	Seq × year 2	-0.409	0.508	-1.414	0.597	0.423
	Seq × year 3	0.831	0.440	-0.041	1.703	0.062
	Seq × year 4	0.750	0.330	0.096	1.405	**0.025**
	SSQ1 baseline	0.984	0.220	0.540	1.429	**<0.0001**
SSQ2	Treatment	-2.319	0.534	-3.380	-1.259	**<0.0001**
	Year 2	0.460	0.472	-0.473	1.392	0.332
	Year 3	0.357	0.426	-0.485	1.200	0.403
	Year 4	0.291	0.336	-0.376	0.959	0.389
	Seq × year 2	-0.999	0.673	-2.328	0.331	0.140
	Seq × year 3	1.824	0.617	0.604	3.044	**0.004**
	Seq × year 4	1.543	0.492	0.567	2.520	**0.002**
	SSQ2 baseline	0.421	0.190	0.039	0.803	**0.032**
SSQ3	Treatment	-1.623	0.550	-2.723	-0.523	**0.005**
	Year 2	-0.288	0.401	-1.081	0.505	0.474
	Year 3	-0.087	0.346	-0.772	0.598	0.802
	Year 4	-0.229	0.259	-0.743	0.284	0.378
	Seq × year 2	0.307	0.565	-0.811	1.425	0.588
	Seq × year 3	1.051	0.494	0.072	2.029	**0.036**
	Seq × year 4	1.205	0.374	0.463	1.947	**0.002**
	SSQ3 baseline	0.473	0.161	0.146	0.800	**0.006**
NCIQ basic	Treatment	-9.620	5.315	-20.218	0.978	0.075
	Year 2	-0.263	4.119	-8.406	7.879	0.949
	Year 3	-1.316	3.601	-8.441	5.810	0.715
	Year 4	-3.863	2.737	-9.292	1.565	0.161
	Seq × year 2	-5.513	5.881	-17.139	6.112	0.350
	Seq × year 3	6.352	5.216	-3.970	16.673	0.226
	Seq × year 4	12.222	4.009	4.273	20.171	**0.003**
	VAS hearing baseline	-19.889	17.165	-54.545	14.767	0.253
NCIQ advanced	Treatment	-3.523	6.426	-16.391	9.345	0.586
	Year 2	-0.376	4.049	-8.383	7.631	0.926
	Year 3	0.764	3.456	-6.076	7.605	0.825
	Year 4	-1.034	2.558	-6.106	4.038	0.687
	Seq × year 2	-3.124	5.787	-14.569	8.322	0.590
	Seq × year 3	2.485	5.006	-7.425	12.394	0.621
	Seq × year 4	5.969	3.747	-1.460	13.398	0.114
	VAS hearing baseline	7.293	21.821	-36.823	51.410	0.740
	HA use	-7.025	9.622	-26.485	12.436	0.470
NCIQ speech	Treatment	0.680	4.581	-8.497	9.857	0.883
	Year 2	-0.340	2.785	-5.848	5.168	0.903
	Year 3	-0.559	2.368	-5.248	4.129	0.814
	Year 4	-1.334	1.746	-4.796	2.128	0.447
	Seq × year 2	-1.014	3.981	-8.888	6.861	0.799
	Seq × year 3	-2.524	3.430	-9.316	4.267	0.463
	Seq × year 4	-1.785	2.558	-6.856	3.286	0.487
	VAS hearing baseline	-29.738	15.691	-61.456	1.980	0.065
	HA use	-4.760	6.921	-18.754	9.234	0.496
NCIQ self esteem	Treatment	-6.806	4.844	-16.488	2.877	0.165
	Year 2	-2.602	3.346	-9.218	4.013	0.438
	Year 3	-2.635	2.873	-8.321	3.051	0.361
	Year 4	-3.684	2.140	-7.928	0.559	0.088
	Seq × year 2	0.713	4.781	-8.741	10.167	0.882
	Seq × year 3	2.618	4.161	-5.618	10.855	0.530
	Seq × year 4	7.084	3.135	0.869	13.299	**0.026**
	VAS hearing baseline	10.330	16.380	-22.770	43.429	0.532
NCIQ activity	Treatment	-6.147	5.595	-17.338	5.044	0.276
	Year 2	-0.150	3.796	-7.656	7.357	0.969
	Year 3	-0.124	3.252	-6.562	6.313	0.970
	Year 4	-2.476	2.417	-7.269	2.317	0.308
	Seq × year 2	-1.719	5.425	-12.447	9.009	0.752
	Seq × year 3	6.108	4.411	-3.217	15.433	0.197
	Seq × year 4	7.802	3.540	0.783	14.822	**0.030**
	VAS hearing baseline	18.473	19.026	-20.007	56.953	0.338
NCIQ social	Treatment	-9.264	4.471	-18.201	-0.327	**0.042**
	Year 2	-0.804	3.149	-7.031	5.423	0.799
	Year 3	0.157	2.711	-5.209	5.523	0.954
	Year 4	1.308	2.025	-2.708	5.325	0.520
	Seq × year 2	0.954	4.499	-7.943	9.850	0.832
	Seq × year 3	5.790	3.927	-1.982	13.563	0.143
	Seq × year 4	7.074	2.966	1.191	12.956	**0.019**
	VAS hearing baseline	15.457	15.016	-14.913	45.827	0.310


*Reference treatment group = simBiCI. Reference year = year 1. The final model contained the following variables: time + treatment + time × treatment + baseline score. In case there was a confounding role for hearing aid use at baseline, this variable was included in the final model as well. VAS, Visual Analog Scale on hearing; SSQ, Speech, Spatial and Qualities of Hearing Scale; NCIQ, Nijmegen Cochlear Implant Questionnaire; HA, hearing aid; SimBiCI, simultaneous bilateral cochlear implantation; seq, unilateral cochlear implantation/sequential bilateral cochlear implantation group. Bold value means statistical significance.*

The SSQ1, SSQ2 and SSQ3 scores were significantly lower in the UCI/seqBiCI group compared with the simBiCI group over time (most evident for SSQ2: -2.32 [-3.38 – -1.26], *p* ≤ 0.001). A significant improvement was seen in the UCI/seqBiCI group after receiving CI2 for the SSQ1 (year 4 vs. year 1: 0.75 [0.10 – 1.41], *p* = 0.025) and the SSQ 2 and 3 (years 3 and 4 vs. year 1, for example year 3 vs. year 1 for SSQ2: 1.82 [0.60 – 3.04], *p* = 0.004). In the simBiCI group, all SSQ scores remained stable in the 4 years of follow-up.

The social interaction score of the NCIQ was significantly lower in the UCI/seqBiCI group compared with the simBiCI group over time (-9.26 [-18.20 – -0.33], *p* = 0.042). Significant increases in basic sound perception, self-esteem, activity and social interaction scores were seen in the seqBiCI group after receiving CI2 (year 4 vs. year 1, most evident for basic sound perception: 12.22 [4.27 – 20.17], *p* = 0.003). In the simBiCI group, all NCIQ scores remained stable in the 4 years of follow-up.

#### Tinnitus Outcomes

**FIGURE 5:**
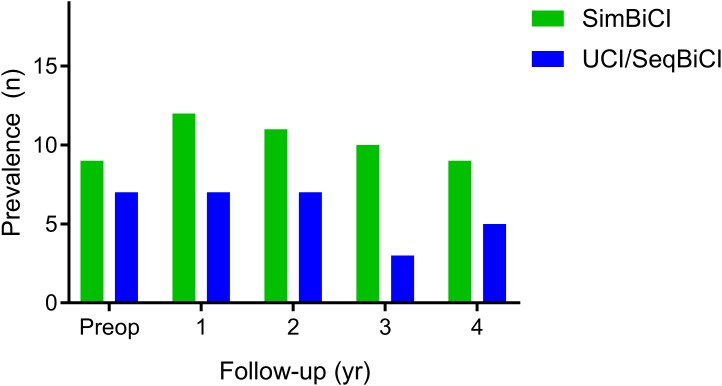
Tinnitus outcomes: a 4-year follow-up. The number of participants with the presence of tinnitus burden. The presence of tinnitus burden is defined as a score higher than 0 on either one of the questionnaires. SimBiCI, simultaneous bilateral cochlear implantation; UCI/seqBiCI, unilateral cochlear implantation/sequential bilateral cochlear implantation group.

Figure 5 shows the prevalence of tinnitus burden preoperatively and during the 4 years of follow-up for both study groups. Although the prevalence appears larger in de simBiCI group, the prevalence of tinnitus burden, corrected for baseline prevalence, did not differ significantly between the UCI/seqBiCI and simBiCI group over time. Also the course of tinnitus burden did not differ between groups and for both groups the presence of tinnitus burden remained stable in the 4 years of follow-up (Table 5).

**Table 5:** Results from a logistic regression analysis with an autoregressive residual covariance matrix (generalized estimating equations type) for the presence of tinnitus burden.

Parameter	Mean	Standard deviation	Odds ratio	Lower bound 95% CI	Upper bound 95% CI	*P*-value
Treatment	-1.110	0.7918	0.330	0.070	1.556	0.161
Year 2	-0.278	0.5155	0.758	0.276	2.080	0.590
Year 3	-0.578	0.6006	0.561	0.173	1.822	0.336
Year 4	-0.869	0.6452	0.419	0.118	1.485	0.178
Seq × year 2	0.637	0.6122	1.891	0.569	6.277	0.298
Seq × year 3	-0.833	0.8966	0.435	0.075	2.520	0.353
Seq × year 4	0.610	0.7606	1.840	0.414	8.172	0.423
Tinnitus baseline	2.404	0.7202	11.068	2.698	45.401	**0.001**


*Reference treatment group = simBiCI. Reference year = year 1. The final model contained the following variables: time + treatment + time × treatment + baseline tinnitus burden presence. SimBiCI, simultaneous bilateral cochlear implantation; seq, unilateral cochlear implantation/sequential bilateral cochlear implantation group. Bold value means statistical significance.*

### Sensitivity Analysis

A sensitivity analysis of the behavioral and self-reported data without participants with missing data revealed no differences regarding direction, effect sizes or significance of the results compared to the primary analysis except for the NCIQ basic sound perception: a significant overall lower NCIQ basic sound perception score was seen in the UCI/seqBiCI group (-11.61, *p*: 0.028). This indicates that missing data in the original analyses did not obscure the results.

### Complications

As shown in Table 6, several complications occurred during the 4 years follow-up period. One participant suffered from vertigo 1 year following simBiCI. Electronystagmographic examination was inconclusive, yet vestibular areflexia was excluded as the cause of vertigo. In one participant in the UCI/seqBiCI group, the left CI had to be explanted and re-implanted 4 years after initial implantation. After initial good performance, the left CI became dysfunctional resulting in an increased stimulation level and coexisting facial nerve stimulation. The cause of this failure remained unclear, imaging and integrity tests were normal. One participant in the simBiCI group with a history of panhypopituitarism, hypothyroidism, kidney failure and systemic lupus erythematosus for which corticosteroids were used, suffered from skin flap necrosis after implantation of the left CI. Surgery was needed to close the subsequent skin defect. One participant suffered from a facial nerve palsy 10 days after seqBiCI (House Brackmann grade 3), of unknown origin, possibly due to a viral infection. The palsy improved spontaneously to House Brackmann grade 2. Another participant in the UCI/seqBiCI group suffered from acute otitis media in the secondly implanted ear for which intravenous antibiotic treatment was needed. One participant in the UCI/seqBiCI group perceived extra sound sensations in CI2. In one participant in the UCI/seqBiCI group, local antibiotics had to be administered to treat a skin infection at the implantation site. In both groups, a participant experienced pain at the ear’s helix due to pressure of the speech processor, for which a support frame and a body worn speech processor were provided. Although it appears that the complication rate is higher in de UCI/seqBiCI group, this was not statistically supported.

**Table 6:** Complications that occurred in this randomized controlled trial during 4 years of follow-up.

Adverse events	SimBiCI	UCI/SeqBiCI	Onset of complication
Vertigo/dizziness	1	0	1 year after simBiCI
Dysfunction of cochlear implant	1	0	4 years after simBiCI
Flap necrosis leading to skin defect	0	1	10 months after seqBiCI
Facial nerve paresis	0	1	10 days after seqBiCI
Acute otitis media	0	1	Two weeks after seqBiCI
Pain	1	1	2 years after simBiCI; 5 months after UCI
Wound infection	0	1	Within 1 month after seqBiCI
Perception of extra sounds	0	1	Within 1 month after seqBiCI


*SimBiCI, simultaneous bilateral cochlear implantation; UCI/seqBiCI, unilateral cochlear implantation/sequential bilateral cochlear implantation.*

## Discussion

### Key Findings

The current RCT evaluated the longitudinal behavioral and self-reported outcomes after simBiCI compared with UCI/seqBiCI, with a 2-year inter-implant interval, in adult patients with severe-to-profound bilateral SNHL with marginal benefit of conventional HAs (an aided CVC phoneme score of ≤50% at 65 dB SPL).

Three participants allocated to the UCI/seqBiCI group, did not proceed to seqBiCI. This study showed that speech perception in noise, localization abilities (SISSS WPS, localization) and self-reported results (SSQ 1 and 2) were significantly worse in the UCI/seqBiCI group compared to the simBiCI group over the course of the 4-year follow-up. This is associated with the poorer results obtained by the UCI/seqBiCI group in the first 2 years of unilateral CI use. In the UCI/seqBiCI group, a significant improvement of these scores was seen after receiving CI2. With this improvement, the UCI/seqBiCI participants (with 2 years of bilateral experience) reached the same level as the simBiCI participants at 3 and 4 years of follow-up.

In one of four QoL questionnaires (TTO), a significantly lower utility score was found in the UCI/seqBiCI group compared to the simBiCI group over time. After the participants received CI2 in the seqBiCI group, their TTO results reached to the level of the simBiCI group. The prevalence of tinnitus burden did not differ significantly between both groups over time.

### Comparison With Literature and Clinical Implication

In previous publications from our group, studying the differences between best performing situation with one CI (with or without a contralateral HA) and simBiCI (Smulders et al., 2016; van Zon et al., 2016), advantages of simBiCI over UCI on spatial speech perception and localization of sounds were demonstrated behaviorally and subjectively. Corresponding to existing observational studies and our previous article from this RCT, the present study identified that patients after seqBiCI also benefit from receiving a second CI as demonstrated in the spatial speech perception and localization of sounds tasks (Ramsden et al., 2005; Summerfield et al., 2006; Zeitler et al., 2008; Olze et al., 2012). Thus, this study shows that after providing deaf patients with one CI, they still benefit from bilateral hearing after sequentially implanting a second CI within an inter-implant interval of 2 years.

No significant differences were found between simBiCI and seqBiCI at 4 years follow-up on behavioral and self-reported outcome measures tested. This finding does not advocate for one of these implantation modalities over the other. Therefore, when considering BiCI, individual factors that might influence the choice for seqBiCI or simBiCI such as for example duration of anesthesia, the intensive rehabilitation, as well as the cost-effectiveness related with each intervention should be taken into account. Yet, delaying implantation of the second ear in our UCI/seqBiCI group did limit hearing in noise and localization capabilities in the 2 years of unilateral CI use. This might have real-life consequences in this timeframe. To our knowledge, the present study is the first to compare outcomes of simBiCI versus seqBiCI in an RCT.

The present findings encourage UCI patients with no or marginal benefit from a contralateral HA to receive a second CI in order to reach improved benefits in speech perception in noise and localization abilities. Thus, there is evidence that implant centers all over the world should consider seqBiCI for all their unilaterally implanted patients with marginal effect or no effect of a contralateral HA. Even though longer duration of inter-implant interval is suggested to cause lesser benefit of CI2 compared to CI1, multiple studies have shown that bilateral results are better than unilateral results (Ramsden et al., 2005; Zeitler et al., 2008). In the present study, the inter-implant interval did not differ between participants in this trial, therefore, the effect of duration of inter-implant interval was not investigated.

It has already been shown that spatial speech perception abilities continue to improve over time for at least 4 years after simBiCI (Eapen et al., 2009; Kraaijenga et al., 2016). Longitudinal results of our study are in line with these findings and show an increased performance in the optimal situation of the SISSS in the simBiCI group over time.

In this study, three participants did not proceed to seqBiCI after UCI. Previous data suggested that not all UCI patients proceed to seqBiCI (Foteff et al., 2016). Therefore, our study finding may be a realistic representation of the actual clinical population at various implant centers. Patients’ withdrawal might be influenced by good performance with CI1, yet conversely, bad performance with CI1 could make patients reluctant to proceed to seqBiCI. Of the three participants in the UCI/seqBiCI group who did not proceed to seqBiCI, two were happy with the results after UCI and one participant who was deafened due to rhesus antagonism had such poor results with UCI that improvement after seqBiCI was not expected.

The lack of overall QoL improvement after seqBiCI in three out of four QoL questionnaires corresponds to earlier findings in literature (Summerfield et al., 2006). QoL questionnaires are commonly used in RCTs to perform a cost-utility analysis. As confirmed by the current study, most general health utility instruments are not appropriate to measure changes after cochlear implantation (Zeitler et al., 2008; Kuthubutheen et al., 2015; Ramakers et al., 2016). For example, the EQ-5D and VAS health instruments do not incorporate a hearing element, and are therefore not sensitive to detect change in QoL as a result of cochlear implantation (Kraaijenga et al., 2017). Moreover, ceiling effects of EQ-5D and TTO were observed, making it even more challenging to detect improvement. Thus, for RCTs on cost-utility analysis, the use of a QoL instrument with a hearing element in cochlear implant studies, for example the HUI3, seems appropriate (Eapen et al., 2009; Kraaijenga et al., 2017). As illustrated in Figure 3, HUI3 scores improved after UCI and simBiCI when compared to the situation before implantation. This finding corresponds to previously published data (Kraaijenga et al., 2017). Nonetheless, to detect smaller differences, such as the additional effect of a second CI in the UCI/seqBiCI group or differences between simBiCI and seqBiCI the HUI3 is not sensitive enough. Compared to QoL questionnaires, QoH questionnaires detected the largest benefit of cochlear implantation, corresponding with previous findings (Summerfield et al., 2006; Olze et al., 2012).

The nature of the complications that occurred during our trial were in line with a previous study, in which vertigo, tinnitus and device failure were among the most reported complications after cochlear implantation (Farinetti et al., 2014). Due to the low sample size, the complication rate could not accurately be compared with literature.

### Strengths and Limitations

The major strength of the current study is the study design. Since allocation bias is excluded, an RCT provides a high level of evidence (level I). Data were prospectively gathered at the same time points for all participants to ensure consistency in reported outcomes. Furthermore, the study design enabled us to examine multiple outcomes: BiCI versus UCI (and bimodal), simBiCI versus seqBiCI and UCI versus seqBiCI. Another strength is the longitudinal method for data analyses (GEE) since it generates more power to detect differences. These strengths add scientific value to knowledge based on previously published studies.

A possible limitation of this study is the relatively small sample size that made it difficult to detect differences in secondary outcomes. The sample size calculation was based on a power analysis aiming at the primary outcome measure. It was performed under the assumption that the increase in power due to repeated measurements would be sufficient for a time by intervention group interaction in the analysis, effectively describing and testing the intervention effect over time. We decided to calculate the sample size in this manner, since a sample size calculation for repeated measurement requires both accurate means (and standard deviations) of the outcome for each time point as well as the correlation (or covariance) between the measurements at different time points. An accurate estimate for especially the correlations was not available at the moment of conception of the trial in 2008. Longitudinal analyses however, have more power compared to cross-sectional analyses because of the repeated observations at the individual level. This approach may have compensated for the lack of power. Three participants were lost to follow-up. This could have led to a bias in treatment effect. However, the sample size calculation incorporated loss to follow-up up to five participants per group. Moreover, sensitivity analyses showed comparable results to the original analyses regarding effect sizes. The localization abilities were scored as percent correct in three different loudspeaker configurations. In retrospect, presenting these results as a mean error in degrees azimuth would be more valuable than a percent correct score. However, our set-up did not allow us to extract mean error data. HAs were not fitted before every test session. In addition, the noise reduction method may have differed per participant. The rehabilitation was done according to the standards of each CI center. Since we used a block randomization per center, possible differences between centers could not have affected the outcome difference between groups. Another possible limitation is the use of logistic regression instead of linear regression for the tinnitus outcomes. Continuous data provide more information than dichotomous data. We only used the presence of tinnitus and not the THI and TQ scores. Since participants not suffering from tinnitus did not complete the questionnaires, the THI and TQ scores of these patients were lacking. Linear regression analysis with all these missing data would result in biased results.

## Conclusion

In this RCT, we evaluated the behavioral and self-reported outcomes after simBiCI compared with UCI/seqBiCI in adult patients with severe-to-profound SNHL with marginal benefit of HAs (aided CVC phoneme score of ≤50%) longitudinally. In the first 2 years of this study, patients after UCI performed significantly worse than patients after simBiCI, on various spatial hearing and localization outcomes. They showed significant improvement after seqBiCI 2 years later and reached the same amount of benefit as the simBiCI group after 4 years of follow-up. Although the interval between sequential implantation was only 2 years, our results show a significant benefit of bilateral implantation both after simultaneous and sequential implantation over UCI with or without a contralateral HA.

## Author Contributions

VK and GR had full access to all the data in the study and take responsibility for the integrity of the data and the accuracy of the data analysis. YS and WG conceptualized and designed the study. VK, GR, YS, AvZ, RF, JF, WH, and RS contributed to the acquisition, analysis, or interpretation of the data. VK and GR drafted the manuscript. YS, AvZ, RS, RF, JF, WH, and WG critically revised the manuscript for important intellectual content. WG received the funding. WG supervised the study.

## Conflict of Interest Statement

JF received non-restrictive grants from Advanced Bionics and MedEl. RF received non-restrictive grants from Advanced Bionics and was sponsored by a neurotological stipendium from the Heinsius Houbolt Foundation. WG received non-restrictive research grants from Cochlear Advanced Bionics and MedEl. The remaining authors declare that the research was conducted in the absence of any commercial or financial relationships that could be construed as a potential conflict of interest.
